# Activating GENeral practitioners dialogue with patients on their Agenda (MultiCare AGENDA) study protocol for a cluster randomized controlled trial

**DOI:** 10.1186/1471-2296-13-118

**Published:** 2012-12-12

**Authors:** Attila Altiner, Ingmar Schäfer, Christine Mellert, Christin Löffler, Achim Mortsiefer, Annette Ernst, Carl-Otto Stolzenbach, Birgitt Wiese, Martin Scherer, Hendrik van den Bussche, Hanna Kaduszkiewicz

**Affiliations:** 1 Institute of General Practice, Medical Faculty, University of Rostock, Doberaner Str. 142, Rostock 18057, Germany; 2 Department of Primary Medical Care, University Medical Center Hamburg-Eppendorf, Martinistr. 52, Hamburg 20246, Germany; 3 Department of General Practice, University of Dusseldorf, Moorenstr. 5, Düsseldorf 40225, Germany; 4 Institute for Biometry, Hannover Medical School, Carl-Neuberg-Str. 1, Hannover 30623, Germany

**Keywords:** Multimorbidity, Primary care, Randomised controlled trial, Polypharmacy, Narrative based medicine

## Abstract

**Background:**

This study investigates the efficacy of a complex multifaceted intervention aiming at increasing the quality of care of GPs for patients with multimorbidity. In its core, the intervention aims at enhancing the doctor-patient-dialogue and identifying the patient’s agenda and needs. Also, a medication check is embedded. Our primary hypothesis is that a more patient-centred communication will reduce the number of active pharmaceuticals taken without impairing the patients’ quality of life. Secondary hypotheses include a better knowledge of GPs about their patients’ medication, a higher patient satisfaction and a more effective and/or efficient health care utilization.

**Methods/design:**

Multi-center, parallel group, cluster randomized controlled clinical trial in GP surgeries. Inclusion criteria: Patients aged 65–84 years with at least 3 chronic conditions. Intervention: GPs allocated to this group will receive a multifaceted educational intervention on performing a narrative doctor-patient dialogue reflecting treatment targets and priorities of the patient and on performing a narrative patient-centred medication review. During the one year intervention GPs will have a total of three conversations à 30 minutes with the enrolled patients. Control: Care as usual. Follow-up per patient: 14 months after baseline interview. Primary efficacy endpoints: Differences in medication intake and health related quality of life between baseline and follow-up in the intervention compared to the control group. Randomization: Computer-generated by an independent institute. It will be performed successively when patient recruitment in the respective surgery is finished. Blinding: Participants (GPs and patients) will not be blinded to their assignment but will be unaware of the study hypotheses or outcome measures.

**Discussion:**

There is growing evidence that the phenomenon of polypharmacy and low quality of drug use is substantially due to mis-communication (or non-communication) in the doctor patient interaction. We assume that the number of pharmaceutical agents taken can be reduced by a communicational intervention and that this will not impair the patients’ health-related quality of life. Improving communication is a core issue of future interventions, especially for patients with multimorbidity.

**Trial registration:**

Current Controlled Trials ISRCTN46272088.

## Background

Multimorbidity can be defined as the coexistence of at least 2 chronic diseases [1]. Care for patients with multimorbidity is a complex task for the GP because of several reasons: Multimorbidity reduces adherence, produces conflicts in treatment decisions due to contra-indications for other diseases, causes polypharmacy and is not supported by clinical guidelines [2]. In caring for patients with multimorbidity a special focus is on coordinating care and integrating different needs of the patient and the medical treatment. A model for optimal care for patients with multimorbidity does not exist. However, the chronic care model [3] opens up a perspective as it highlights elements that are necessary for optimal chronic illness care on the level of the single patient. These elements relate to [4]:

• the joint definition of problems by patient and physician,

• targeting, goal setting and planning,

• a continuum of self-management training and support services, and

• active and sustained follow-up by the professional.

Up to date it is not known how these elements can be realized in practice. The few intervention studies carried out for improving outcomes in patients with multimorbidity in primary care and community settings involved complex interventions with multiple elements including also changes to the organisation of care delivery. The results suggest that interventions to date have had mixed effects but have shown a tendency to improve prescribing and medication adherence [5]. Other studies focused on interventions to improve appropriate polypharmacy. In a systematic review Patterson et al. concluded that it is unclear if the interventions studied resulted in a clinically significant improvement, but they appeared beneficial in terms of reducing inappropriate prescribing and medication-related problems [6].

It is also not known which specific aspects of care for patients with multimorbidity by German GPs can and should be optimized within the available resources and which of them have a realistic chance to show sustained effects. What we do know is that German GPs see their patients very often for a short time at each visit. German doctors in ambulatory care see 243–309 patients a week, much more than e.g. UK doctors with 154–205 contacts. It is understood that this number includes a number of patients without face to face contact (e.g. repeated drug prescriptions [7]). This high contact frequency results in a contact duration of only 7.6-7.8 minutes per patient [8,9]. These and other research results [10] suggest that the elements of chronic illness care mentioned above may insufficiently be put into practice as they afford a considerable amount of communication time. Routine doctor-patient consultation is usually determined by real and by perceived time-constraints and a by doctor-centred consultation-style.

The concept of “narrative based medicine” was established by Greenhalgh et al. [11] in order to stop this negative development. The concept assumes that patient narratives of illness can provide a framework for approaching a patient’s problems holistically and may uncover diagnostic and therapeutic options. This concept has not yet been linked to the Chronic Care Model.

The MultiCare AGENDA study investigates the efficacy of a complex multifaceted intervention aiming at increasing the quality of care of GPs for patients with multimorbidity. When treating patients with multimorbidity it is crucial to prioritize and set goals. Only if the patient perspective is known, the main focus of treatment can be defined. Therefore in its core, the intervention aims at enhancing the doctor-patient-dialogue and identifying the patient’s agenda and needs, including the need for social support. Also, a medication check is embedded in this intensified dialogue. Our primary hypothesis is that a more patient-centred communication can disclose the main focus of treatment of the patient which often will have implications for medication use. Therefore the primary hypothesis is that the patient-centered communication in the form of narrative based medicine leads to a reduction of the number of medication taken without reducing health related quality of life.

### Rationale of the hypothesis

In a pilot study [12] we developed and evaluated an intervention package that is based on constitutional elements of the Chronic Care Model and the concept of narrative based medicine as presented above.

In a first step we conducted qualitative in-depth interviews with 10 GPs and two of their multimorbid patients respectively. We found that GPs and their multimorbid patients pursued different agendas. Patients focused on restrictions in their activities of daily living and their fear of becoming dependent on others. The GPs knew about these restrictions variably well but focussed rather on medical diagnoses and risk factors of their patients. Patients and GPs appreciated very much the opportunity to meet each other regularly. Narrative interviews were perceived as a good opportunity to reflect about the treatment experiences and they were experienced by both as a good stimulus to address certain issues at the next meeting. Surprisingly, in this pilot study polypharmacy was only a side issue for both patients and GPs although the phenomenon was apparently existent. We concluded that there is a need for a regular revision and readjustment of treatment goals between the GP and his patient. Regular consultations outside the normal routine, scheduled twice a year, may be helpful for GPs and their patients. A narrative based approach might be a better way of identifying problems and the agenda of the patient than e.g. predefined problem check lists etc. Also, it might be important to draw the attention of the patient and his GP on polypharmacy by means of a comprehensive but still “attractive” narrative medication check.

The concept of an intervention based on these conclusions was positively evaluated within focus group discussions in Hamburg and Dusseldorf. The majority of participating GPs were quite enthusiastic about the suggested interventional elements. The focus groups showed the general acceptability of the suggested intervention and helped to “fine tune” some elements for the final intervention concept.

In a second step we conducted a feasibility study with 16 GPs in Hamburg and Dusseldorf and 135 patients with multimorbidity. Additional 5 GPs participated in the first training on narrative-based communication but then, due to time constraints, did not recruit any patients and were excluded from the study. The participating GPs were trained in narrative-based communication and narrative medication review and performed the scheduled consultations within the project as planned. We could show the feasibility of the elaborated concept, both the concept of the intervention itself and the study design.

## Methods/design

### Design

MultiCare AGENDA is designed as a two-armed cluster-randomized controlled trial in general practice. The study will evaluate the efficacy of structured, narrative-based, doctor-patient-dialogues in patients with multimorbidity. Given that to date no clinical trial has investigated the efficacy of this approach the narrative based intervention must be tested against care as usual.

### Intervention

GPs allocated to the intervention arm will receive an educational intervention: (1) on performing a narrative doctor-patient-dialogue reflecting treatment targets and priorities of the patient, and (2) on performing a narrative patient-centred medication review.

During the one year intervention GPs will have a total of three conversations with the enrolled patients instead of routine consultations. Each conversation is scheduled for about 30 minutes. The first conversation will focus on treatment targets and priorities of the patient, the second will focus on the medication taken by the patient and the third will combine the elements of both previous conversations.

The idea behind the approach of installing a structured framework of regular consultations is that this will eventually reduce the high number of (often unscheduled) consultations. The concept of narrative medicine shall facilitate the development of the patient’s own agenda.

Follow-up per patient: 14 months after the baseline interview; duration of intervention per patient: 12 months; all additional treatments are allowed.

GPs of the control group will perform care as usual and at the end of the intervention they will be offered to participate in a similar educational intervention as in the intervention group given. This offer will outweigh the possible lower level of motivation of these GPs of having been randomised to the control arm. GPs of the control group will also be financially recompensated equally to the GPs of the intervention group.

### Study population and recruitment

The study will be conducted in three larger German cities, i.e. Hamburg, Dusseldorf and Rostock. In each study centre GPs will be randomly selected from the register of the Association of Statutory Health Insurance Physicians and asked by mail to participate in our study. Additionally, we will recruit GPs using advertisements in internet and print media.

Inclusion criteria for GPs are:

• willingness to participate in the study regardless of randomisation to the intervention or control arm,

• establishment of an own GP surgery for 2 years at least, and

• usage of practice software that is able to create a total list of patients based on age.

Exclusion criteria for GPs are participation in our feasibility study or the MultiCare Cohort Study [13].

Participating GPs will retrieve a list of all patients aged between 65 and 84 years who have consulted them within the last completed quarter (i.e. 3 month period). From this list 25 patients will be selected randomly (using random number tables), checked for exclusion criteria and – if eligible for the study – invited to participate in the study by a letter from their GP. In case of interest, the patient will consult the GP and receive written and verbal information. The information covers aims and procedures of the study, selection of participants, data collection, processing and storage as well as possibilities for cancellation. In case of acceptance, participants will have to sign an informed consent form to participate in the study.

Exclusion criteria for patients are:

• Poorly known patients to the GP because of accidental consultation,

• Patients known by the GP for less than 12 months,

• Insufficient ability to consent (e.g. dementia),

• Insufficient ability to participate in interviews (e.g. psychic illness),

• Severe illness probably fatal within 3 months according to the GP,

• Residence in a nursing home,

• Deafness,

• Insufficient ability to speak and read German, and

• Participation in other scientific trials.

Inclusion criteria: Only patients with at least 3 chronic conditions out of a list of 42 diagnosis groups will be included in the study. This list includes the following diagnosis groups:

• Severe vision reduction [H17-H18, H25-H28, H31, H33, H34.1-H34.2, H34.8-H34.9, H35-H36, H40, H43, H47, H54],

• Joint arthrosis [M15-M19],

• Diabetes mellitus [E10-E14],

• Chronic ischemic heart disease [I20-I21, I25],

• Thyroid dysfunction [E01-E05, E06.1-E06.3, E06.5, E06.9, E07],

• Cardiac arrhythmias [I44-I45, I46.0, I46.9, I47-I48, I49.1-I49.9],

• Obesity [E66],

• Purine/pyrimidine metabolism disorders/Gout [E79, M10],

• Prostatic hyperplasia [N40],

• Lower limb varicosis [I83, I87.2],

• Liver diseases [K70, K71.3-K71.5, K71.7, K72.1, K72.7, K72.9, K73-K74, K76],

• Depression [F32-F33],

• Asthma/COPD [J40-J45, J47],

• Noninflammatory gynaecological problems [N81, N84-N90, N93, N95],

• Atherosclerosis/PAOD [I65-I66, I67.2, I70, I73.9],

• Osteoporosis [M80-M82],

• Renal insufficiency [N18-N19],

• Cerebral ischemia/Chronic stroke [G45, I60-I64, I69],

• Cardiac insufficiency [I50],

• Severe hearing loss [H90, H91.0, H91.1, H91.3, H91.8, H91.9],

• Chronic cholecystitis/Gallstones [K80, K81.1],

• Somatoform disorders [F45],

• Hemorrhoids [I84],

• Intestinal diverticulosis [K57],

• Rheumatoid arthritis/Chronic polyarthritis [M05-M06, M79.0],

• Cardiac valve disorders [I34-I37],

• Neuropathies [G50-G64],

• Dizziness [H81-H82, R42],

• Urinary incontinence [N39.3-N39.4, R32],

• Urinary tract calculi [N20],

• Anemias [D50-D53, D55-D58, D59.0-D59.2, D59.4-D59.9, D60.0, D60.8, D60.9, D61, D63-D64],

• Anxiety [F40-F41],

• Psoriasis [L40],

• Migraine/chronic headache [G43, G44],

• Parkinson’s disease [G20-G22],

• Cancers [C00-C26, C30-C41, C43-C58, C60-C97, D00-D09, D37-D48],

• Allergies [H01.1, J30, K52.2, K90.0, L23, L27.2, L56.4, T78.1, T78.4, T88.7],

• Chronic gastritis/GERD [K21, K25.4-K25.9, K26.4-K26.9, K27.4-K27.9, K28.4-K28.9, K29.2-K29.9],

• Sexual dysfunction [F52, N48.4],

• Insomnia [F51, G47],

• Tobacco abuse [F17], and

• Hypotension [I95].

The criterion of at least three chronic diseases or disease complexes out of a predefined list was chosen in order to include patients, for whom the diseases represent a certain burden and who we can expect to use a considerable amount of medication [14]. Preliminary data of the baseline assessment in the MultiCare Cohort Study [9] show that patients included along the above listed criteria averagely use 7.26 medications (median 7). Hence there is potential for reduction. The exclusion criteria shall ensure that patients included into the study are able to actively participate in the intervention. The study population will be representative of patients with multimorbidity in primary care who can communicate with their GP.

### Randomization

The randomization will be carried out as a cluster randomization, in which the GPs will be allocated either to the intervention or to the control group. Randomization will be performed successively when patient recruitment in the respective surgery is finished. It will be performed by an experienced independent institute (Institute for Biometry, Hannover Medical School).

### Primary outcome measures

The first primary outcome measure is the number of pharmaceutical agents taken by the patients. This measure will be assessed as a part of the patient interview in form of a complete medication survey. The interviewer will collect data on all pharmaceutical products used by the patient within the last twelve months. The data include product name, pharmaceutical form, German national drug code (“Pharmazentralnummer” - PZN -), periodic or prn (pro re nata) medication, dosage and frequency (for periodic medication). The interviewer will ask the patient to show the packages of the pharmaceuticals to get the most valid information. If product name and drug code are not available, the patient will be asked for the medical indication of the drugs.

The second primary outcome measure is health related quality of life as measured by the EQ-5D [15]. These data will also be collected as a part of the patient interview.

### Study hypotheses

We assume that the number of pharmaceutical agents taken by the patient can be reduced in the intervention group. We will compare the change in medication intake between baseline and follow-up in the intervention and control group. We expect that the mean difference between the changes in both groups will be at least 1.5 drugs less in the intervention group. A minimum difference of 0.5 drugs between both groups is defined as clinically relevant.

It is assumed that a reduction of medications used will not impair quality of life. We will compare the change in health related quality of life between baseline and follow-up in the intervention and control group. We expect that the mean change in the intervention group will not be statistically significantly inferior to the mean change in the control group.

### Secondary outcome measures

We will assess the following secondary outcome measures:

a. GP’s knowledge about the medication taken by the patient (including GP’s own prescriptions, prescriptions of other specialists, and use of over the counter drugs). We will assess the medication documented in GP charts at baseline and follow up and compare it with the medication data collected in patient interviews using similarity measures as the Jaccard coefficient [16]. We expect that the GP’s knowledge will increase due to our intervention.

b. Patient satisfaction with GP services as measured by the EUROPEP [17] and patient empowerment as measured by the Health Care Empowerment Questionnaire [18], both collected in patient interviews. We assume that patient satisfaction and empowerment will improve.

c. Health care utilization and costs, assessed in a comprehensive way by the Leipzig Supply and Cost Instrument [19] in patient interviews as well as number of contacts with the GP and GP’s referrals to specialists assessed in GP interviews.

### Methods against bias

Selection bias will be minimised by a standardised recruitment procedure (chart registry), performed by trained research assistants. In order to collect the required data for a CONSORT flow chart (for clustered trials), the full recruitment process will be documented.

#### **Public registration**

Before start, the study was registered in a public internet trial archive (ISRCTN46272088).

#### Blinding

Participants (GPs and patients) will not be blinded to their assignment (which is practically not possible) but will be unaware of the study hypotheses or primary outcome measures.

#### Standardization of assessments

To ensure high data quality assessors will be trained on standardised patients. The personnel of the participating practices will not be involved in the collection of data.

#### Monitoring

An independent monitor (Institute for Biometry, Hannover Medical School) will conduct data monitoring to ensure high quality data in adherence to the study protocol.

#### Detection bias

The statistical analysis of endpoints will be performed by a statistician blinded to group assignment.

GPs of the control group will be offered the two training sessions at the end of the intervention in order to avoid a possible lack of motivation for recruitment and documentation. Furthermore they will get the same financial incentive as the GPs of the intervention group.

### Stopping rules

As the planned intervention does not introduce any specific therapeutic changes but focuses at intensifying the doctor-patient relation and over-thinking of therapeutic aims negative effects are very unlikely. However, the following stopping rule will be implemented and monitoring will cover these events at group level:

• if the average rate of hospital admissions increases more than 30% in any one group.

### Sample size/power calculations

For the sample size estimation of both endpoints an α=0.025 is assumed in order to adjust for multiple testing. The sample size calculation is based on preliminary analyses of the baseline assessment of the MultiCare Cohort study, which used the same inclusion criteria that are scheduled for this trial and also recruited patients via GP surgeries. In the MultiCare Cohort study patients averagely used 7.26 ± 3.5 medications (Median 7). We expect a mean reduction of 1.5 drugs per patient in the intervention group (difference baseline – follow-up) and no change (mean reduction 0) in the number of drugs in the control group (effect-size: 0.429). A common standard deviation of 3.5 drugs is assumed. Based on the data of the MultiCare Cohort study the intracluster correlation is ρ=0.14 and a minimum of 10 patients per GP are targeted. Under these assumptions and with α=0.025 and a power of 80% totally 48 GPs (475 patients) are necessary to detect the expected difference (2-sided t-test for equal variances). Given a drop-out rate of 20%, 594 patients (and 60 GP surgeries) are needed altogether. The dropout rates in other multicenter studies of the principal investigators are comparable (e.g. AgeCoDe: 16% within 1.5 years).

Regarding the other primary endpoint it is assumed that health related quality of life will not be inferior in the intervention group compared with the control group at the end of the intervention. On the instrument used (EQ-5D value set UK [20]) -0.59 till 1.0 points can be achieved. At the baseline assessment of the MultiCare Cohort study patients scored 0.68 ± 0.30 points. For testing non-inferiority it is assumed that the mean score of the intervention group at the end of the intervention is at most half a standard deviation (i.e. 0.15 points) less than that of the control group. Given a standard deviation of 0.30, an α=0.025, a power of 80%, an intra-cluster correlation of ρ=0.14, 128 patients are needed (1-sided test). Considering a design effect of 2.26 (minimum of 10 patients per GP) and a dropout rate of 20% we would need 362 patients altogether. As this number of patients is smaller than that for the other primary endpoint, the inclusion of 600 patients will be sufficient also for this health related quality of life endpoint. Statistical analyses will be on the intention to treat basis.

### Statistical analyses

Because of the cluster randomisation hierarchic multi-level models with GP as random effect (e.g. mixed model analysis of variance) will be applied for the statistical analyses of primary and secondary endpoints. Possible baseline imbalances and confounding variables will be controlled by adjustment. The data will be analysed according to the intention-to-treat principle. Because of the two primary endpoints Bonferroni correction will be applied to adjust for multiple comparisons.

### Quality assurance and safety

Reliability trainings and checks will be performed before starting the study and subsequently yearly with the whole staff involved in interviewing and data collection.

IT, data management and quality assurance will be provided by the Institute of Biometry, Hannover Medical School. Quality assurance consists of procedures for prevention of insufficient data quality, detection of inaccurate or incomplete data and action to improve data quality, e.g. user training sessions, automatic plausibility and integrity checks within the remote data entry system and data error reports for the local centres. In addition the centres will regularly receive feedback by quality reports. Two monitoring and training visits per centre are planned: one at the beginning of the trial and one after finishing the recruitment phase. In addition a random sample of paper questionnaires (5%) will be compared with the data entries in the database. Adverse events will be monitored and reported.

### Ethics approval

The study was approved by the Ethics Committee of the Medical Association of Hamburg in July 2011 (Approval-No. PV3788).

## Discussion

### Justification of primary outcome measures

On the one hand, the number of pharmaceutical agents taken shall be at a minimum with respect to patient safety (the less non-essential medication, the lower the risk of adverse effects and interactions) and health care costs. On the other hand, necessary drugs should be used reliably by the patients. This is often not the case as the average rate of non-adherence to chronic medication has been estimated at about 50% [21]. According to Spinewine et al. [22] “the evidence is mixed and contradictory that inappropriate prescribing, defined by process measures, is associated with adverse patient outcomes.” However, this may be largely due to important limitations in the methods of the studies reviewed. Therefore, despite the poor evidence, there is a consensus that reduction of harmful drug load and reliable use of needed drugs are of great importance, especially in patients with multimorbidity [23].

There is growing evidence that the phenomenon of polypharmacy and low quality of drug use is substantially due to mis-communication (or even non-communication) in the doctor patient interaction [24,25]. Also, agenda divergence may lead to more referrals to (or self directed use of) specialists with further drug prescriptions, of which the GP may not be informed. The problem of specialists prescribing medication without informing the GP is typical for Germany and some other countries in which there is practically free access to ambulatory working specialists. Also, Spinewine et al. [19] highlight communication as a core of future interventions: “Although several studies addressed communication between different health-care providers through multi-disciplinary approaches, we believe the issue of communication between prescribers and their patients has been overlooked. (…) The involvement of patients or their carers in decision making relevant to prescribing is a real challenge, especially in a frail elderly population. However, this approach seems promising.”

For these reasons the number of pharmaceutical agents taken was chosen as a primary endpoint, which – in contrast to subjective measures - can be collected with high reliability. We assume that the number of pharmaceutical agents taken can be reduced. However, in order to include a measure that reflects the subjectivity of the patient we will also measure the health related quality of life. It is assumed that a reduction of medications used will not impair quality of life.

### Justification of secondary endpoints

Further we expect that the intervention will influence subjective measures like patient satisfaction with the services of the GP or patient empowerment. The instruments chosen for measuring the secondary endpoints are all validated and frequently used in high quality studies. However, subjective measures are not widely acknowledged as good primary endpoints as they always show validity problems. Therefore these parameters were chosen as secondary endpoints.

We also expect that the intervention will increase efficiency and effectiveness of health care utilization. On the one hand, health care costs could be reduced due to a reduction of medication, due to focussing on treatment goals that are important for the patient (and dropping treatments that are not relevant in this context) or due to gaining better disease control through regular encounters with less unplanned hospital admissions (higher efficiency). On the other hand costs could rise as a result of so far unmet health problems and needs of support, e.g. if nursing services are required (higher effectiveness). Therefore health care utilization and costs will be assessed in a comprehensive way by the Leipzig Supply and Cost Instrument, which already is used successfully in the MultiCare Cohort Study [9] and the AgeCoDe-study [16].

## Conclusions

The MultiCare AGENDA study will show if enhancing the doctor-patient-dialogue and identifying the patient’s agenda and needs can increase the quality of care for patients with multimorbidity. We presume that our intervention can lower the number of pharmaceuticals without impairing the patients’ quality of life. Other possible effects include a better knowledge of GPs about their patients’ medication, a higher patient satisfaction and a more effective and/or efficient health care utilization.

## Competing interests

The authors declare that they have no competing interests.

## Authors’ contributions

HK and AA initiated and designed the study, further development was performed by all authors. The paper was drafted by IS and revised by all authors. All authors read and approved the final manuscript.

## Pre-publication history

The pre-publication history for this paper can be accessed here:

http://www.biomedcentral.com/1471-2296/13/118/prepub
